# Inhibition of *Francisella tularensis* phagocytosis using a novel anti-LPS scFv antibody fragment

**DOI:** 10.1038/s41598-019-47931-w

**Published:** 2019-08-06

**Authors:** Adva Mechaly, Uri Elia, Ron Alcalay, Hila Cohen, Eyal Epstein, Ofer Cohen, Ohad Mazor

**Affiliations:** 1 The Department of Infectious Diseases, Israel Institute for Biological Research, Ness-Ziona, Israel; 2 The Department of Biochemistry and Molecular Genetics, Israel Institute for Biological Research, Ness-Ziona, Israel; 3 The Department of Biotechnology, Israel Institute for Biological Research, Ness-Ziona, Israel

**Keywords:** Bacterial infection, Pathogens

## Abstract

*Francisella tularensis* (*Ft*), the causative agent of lethal tularemia, is classified as a category A biological warfare threat agent. While *Ft* infection is treatable by antibiotics, many failed antibiotic treatments were reported, highlighting the need for effective new treatments. It has been demonstrated that binding of antibody-coated bacteria to the Fc receptor located on phagocytic cells is a key process needed for efficient protection against *Ft*. Yet, *Ft* utilizes the same receptor to enter the phagocytic cells in order to escape the immune system. To address the question whether an anti-*Ft* LPS antibody lacking the ability to bind the Fc receptor may inhibit the entry of *Ft* into host cells, a soluble scFv (TL1-scFv) was constructed from an anti *Ft*-LPS antibody (TL1) that was isolated from an immune single-chain (scFv) phage-display library. Bacterial uptake was assessed upon infection of macrophages with *Ft* live attenuated strain (LVS) in the presence of either TL1 or TL1-scFv. While incubation of LVS in the presence of TL1 greatly enhanced bacterial uptake, LVS uptake was significantly inhibited in the presence of TL1-scFv. These results prompt further experiments probing the therapeutic efficacy of TL1-scFv, alone or in combination with antibiotic treatment.

## Introduction

*Francisella tularensis* (*Ft*), the causative agent of lethal tularemia, is a virulent Gram-negative, facultative intracellular bacterium. Due to its high infectivity and mortality rates, *Ft* is classified as a category A biological warfare threat agent by the CDC^1^. Tularemia is usually treatable by antibiotics, however, only few are recommended as the treatment of choice^2^. Moreover, there are natural strains of *Ft* that acquired antibiotic resistance (for example to beta-lactams and colistin)^3,4^. In addition, many therapeutic failures and relapses of infected patients were reported^5,6^ with about 2% mortality rates of antibiotic-treated patients^7^. Thus, several approaches were made to develop novel and effective treatments for tularemia^8^.

The role of antibody-mediated protection against intracellular pathogens in general and *Ft* in particular has long been controversial. For example, several studies have shown that antibodies directed against the LPS of *Ft* can be used for the treatment of mice infected with *Ft* attenuated strain (LVS) and not for those infected with the virulent strain (type A SchuS4)^9–11^. While *Ft* utilizes several receptors, including Fc receptor (FcγR) to enter the cytosol and escape from the immune system^12–15^, binding of antibody-coated bacteria to the same receptor is a key process needed for efficient protection against LVS^9,16^. Interestingly, this exact uptake mechanism is also being investigated as a way to enhance the uptake of inactivated *Ft* in order to provoke efficient immune response and as a mean to create a platform for vaccination^17^.

It was previously suggested that the failure of anti-*Ft* antibodies to provide efficient protection against the virulent strain, although they can bind it very efficiently, is due to a complete shutdown of the inflammatory response needed for efficient antibody-mediated clearance of the bacteria^10^. Yet, others have shown that opsonization of the SchuS4 strain using antibodies changed the intracellular fate of the bacteria and limited its ability to replicate in the cytosol^18^. As the recognition of *Ft* at the host cell membrane is a key step in the infection process, we asked whether the creation of an anti-*Ft* LPS antibody that lacks the ability to bind to the FcγR will inhibit the entry of *Ft* into the host cell. In order to create a specific and high-affinity antibody, it was decided to incorporate an immunization methodology that promotes high affinity antibodies *in vivo*, together with efficient screening methods using phage-display libraries. This practice was successfully applied previously, resulting in the isolation of potent and high-affinity antibodies against ricin and abrin from immunized non-human primates and rabbits, respectively^19,20^. In this work we report the isolation of an anti-*Ft* LPS antibody that reduced bacterial uptake by cultured macrophages.

## Results and Discussion

### Immunization and characterization of the antibody response

**Figure 1:**
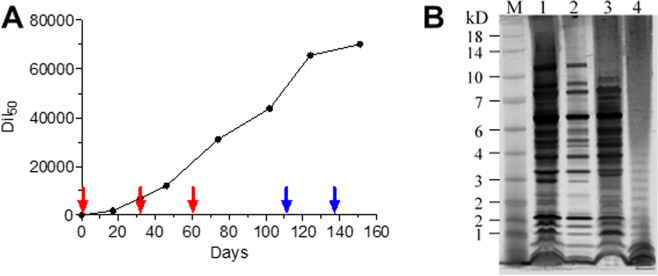
Characterization of the elicited polyclonal anti-*Ft* antibodies. (**A**) Monitoring of anti-*Ft* polyclonal antibodies development during rabbit immunization. The rabbit was injected with sub-cutanic injections of 1 × 10^8^ CFU (red arrows) or 1 × 10^9^ CFU (blue arrows). Antibody titer was determined by ELISA using LVS as the coated layer. (**B**) Western blot analysis of the elicited antibodies. M - Protein size marker; 1 - LVS lysate; 2 - LVS-S lysate; 3 - SchuS4 lysate; 4 - purified LPS of LVS.

Previous studies have demonstrated that the combination of an efficient immunization protocol with proficient screening methods, results in the isolation of potent antibody clones^19,20^. Accordingly, we hypothesized that in order to isolate highly specific antibodies toward the *Ft* LPS moiety, the immunization process should be carried out with live bacteria. Since rabbits are a vector of tularemia and are naturally susceptible to this pathogen, the immune reaction following infection was studied previously by evaluating anti-bacteria antibody titers, changes in clinical and hematological parameters and more^21–23^. Others have also further analyzed the antibody responses towards specific proteins and towards the LPS moieties^24,25^. Here, we took advantage of the fact that rabbits can tolerate infection with live LVS^26^ in order to generate an immunization protocol that involved repeated exposures of rabbits to this strain, in order to elicit a strong immune response. To this end, a female rabbit was infected with three successive injections of 1 × 10^8^ CFU LVS and the elicited titer against the whole bacteria was evaluated and presented as the half dilution value (Dil_50_) corresponding to 50% of the maximal binding of the animal serum towards the coated antigen (Fig. 1A). Interestingly, the antibody titer continued to increase within the following 40 days post injection, thus raising the possibility that LVS was still present at that time point. To increase the anti-LVS titer, the animal was further exposed to two successive high doses of LVS (1 × 10^9^ CFU) until a plateau was reached.

To evaluate the pattern of the elicited antibodies toward *Ft*, a Western Blot analysis was performed using bacterial lysates (Fig. 1B). We first evaluated the reaction of the elicited antibodies against a purified LPS fraction, extracted from LVS bacteria (lane 4). The resulting binding-pattern (characterized by the ladder pattern) indicated that at least part of the response was raised against the LPS moiety. An examination of the response against whole LVS bacterial lysate (lane 1) indicated that the elicited antibodies recognized also other, non LPS components of the bacteria. To further confirm these findings, the antibodies were reacted with LVS-S lysate (lane 2), a phase variation of *Ft* that has impaired O-antigen synthesis (which resembles the LVS gray variant^27,28^). As expected, the antibody binding pattern lacks the characteristic LPS ladder while demonstrating a strong binding to other bacterial proteins. Importantly, a similar binding-pattern was observed toward SchuS4 extracts.

### Isolation of anti-LPS high affinity antibody

A final injection of LVS (1 × 10^9^ CFU) at day 165 was administered to the immunized animal and a scFv phage-display library was constructed from cDNA templates derived from RNA isolated from its spleen, bone marrow and peripheral blood taken at day 172. Following three rounds of panning using plate-coated LVS bacteria, individual clones were screened by direct phage-ELISA for their ability to bind LVS. It was found that 80% of the colonies reacted with LVS, and were all found to possess the same VH-VL sequence.

**Figure 2:**
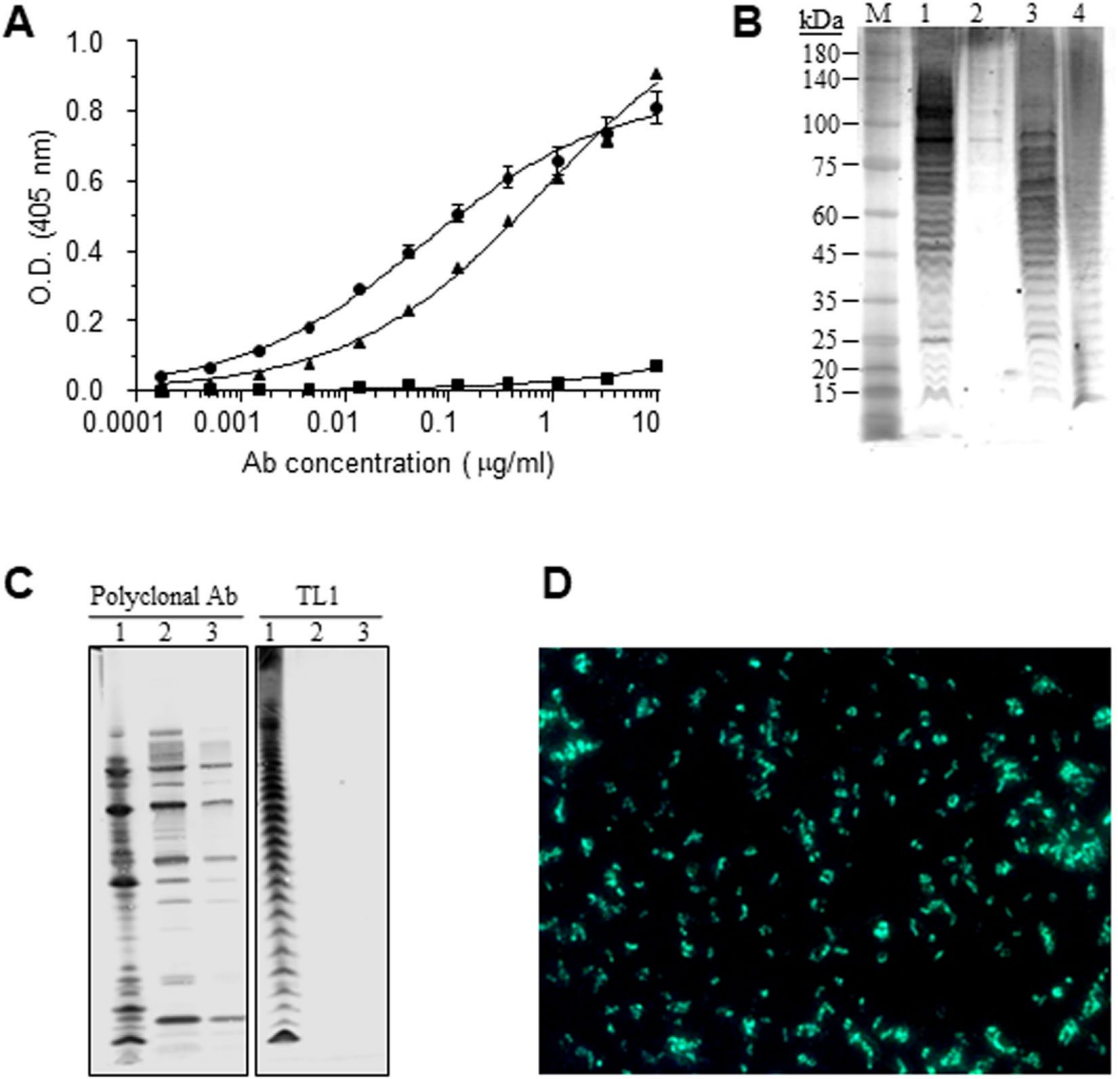
Binding characterization of TL1. (**A**) The reactivity profile of TL1 was determined by ELISA using either LVS (circles), SchuS4 (triangles) or LVS-S (squares) as the adsorbed layer. Points are the mean ± STD of quadruplicates. (**B**) Western blot analysis of TL1 reacted with: 1 - LVS lysate; 2 - LVS-S lysate; 3 - SchuS4 lysate; 4 - purified LPS of LVS. M - Protein size marker; (**C**) Western blot analysis of anti-*Ft* polyclonal antibodies or TL1 reacted with: 1 – LVS lysate; 2 – *Fp* strain 25015 lysate; 3 – *Fp* strain 25017 lysate. (**D**) Immunofluorescence assay of LVS using Alexa 488-conjugated TL1.

The isolated scFv-displayed antibody was reformatted and expressed as a chimeric antibody^20,29^ composed of rabbit variable chains and human constant regions (IgG1/*κ*). The novel antibody, termed TL1 was expressed in cultured cells and then further characterized for its ability to bind the target bacteria. Using ELISA, it was found that TL1 binds both LVS and SchuS4 with high affinity, while it does not bind the LVS-S strain (Fig. 2A), suggesting that it is specific to the *Ft* O-antigen. Western blot analysis also confirmed that observation, where TL1 reacted solely with LVS, SchuS4 or purified LPS (Fig. 2B). According to a previous report that characterized the binding pattern of several anti-*Ft* LPS mAbs^30^, it can be assumed that TL1 binds the longer chains of the LPS ladder and more specifically the four-sugar repeats in the LPS O-antigen chains.

The specificity of this antibody toward *Ft* was further confirmed using two strains of the closely related bacteria *Francisella Philomiragia* (*Fp*). Indeed, while control anti-*Ft* polyclonal antibodies recognize *Fp*, TL1 does not recognizes this species (Fig. 2C). In addition, in ELISA binding assays where other gram-negative bacteria (including *Y*. *pestis* and *S*. *typhimurium*) were used, TL1 did not recognize the above species, thus further indicating its high specificity (data not shown).

The binding of TL1 to LVS was also analyzed by an immunofluorescence assay (IFA), where it exhibited the LPS characteristic staining as would be expected from an anti-LPS antibody (Fig. 2D).

**Figure 3:**
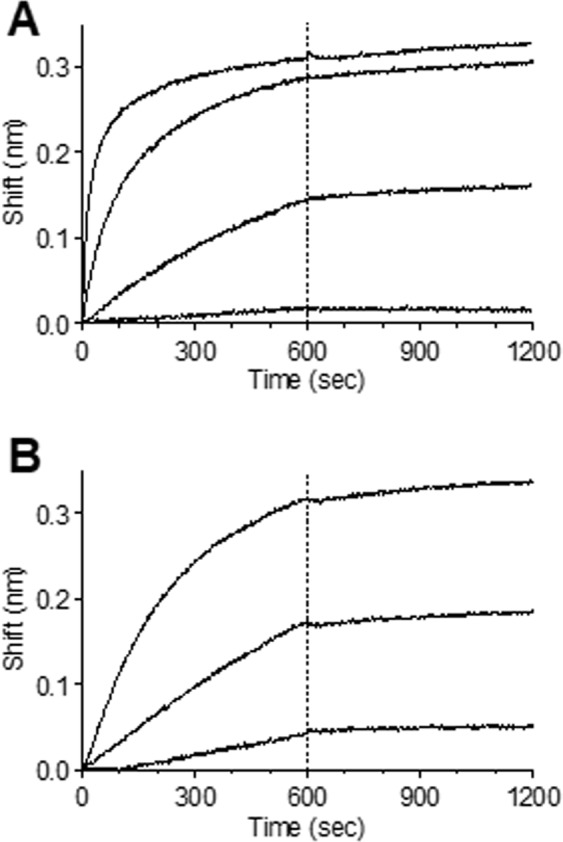
Affinity measurements. The binding kinetics of TL1 were measured using the Octet Red system. Biotinylated TL1 was immobilized on the sensor and reacted for 600 s with increasing concentrations of (**A**) LVS (from bottom up:1 × 10^6^, 1 × 10^7^, 1 × 10^8^ and 1 × 10^9^ CFU/ml) or (**B**) purified LPS (from bottom up: 0.2, 1 and 5 μg/ml). The sensors were then immersed in buffer for another 600 s (marked by dashed line).

To further characterize the binding of TL1 to *Ft*, we used the Octet Red biolayer interferometry system. The binding profile of TL1 with different concentrations of LVS revealed a positive dose response where at higher LVS concentrations a faster association and saturation was achieved (Fig. 3A). Accurate determination of antibody affinity requires the interaction of the antibody with several concentrations of the antigen. Here, due to the repetitive nature of the target antigen of TL1, it is impossible to calculate its concentration and therefore the association constant (*k*_on_) could not be determined. On the other hand, the dissociation constant (*k*_off_) does not require prior knowledge of the antigen concentrations and therefore can be calculated. It was found that the dissociation rate was extremely slow, below 1 × 10^−7^ s^−1^ which is the Octet Red detection limit, indicating that TL1 exhibits high affinity that is probably in the sub-pM range. Moreover, no dissociation between TL1 and LVS was observed even at a highly acidic environment (pH 2.7).

To further strengthen this observation, the binding assay was repeated using several concentrations of purified LPS (the exact molarity cannot be determined due to the high variability of the LPS chains length within the sample) and indeed, a similar binding pattern was observed and no dissociation could be detected (Fig. 3B). This phenomena (antibodies exhibiting sub-pM affinities) was previously reported by our group for several antibodies against ricin, that were isolated from phage-display libraries originated from immunized non-human primates^20^.

### Sensitive detection of *F. tularensis*

**Figure 4:**
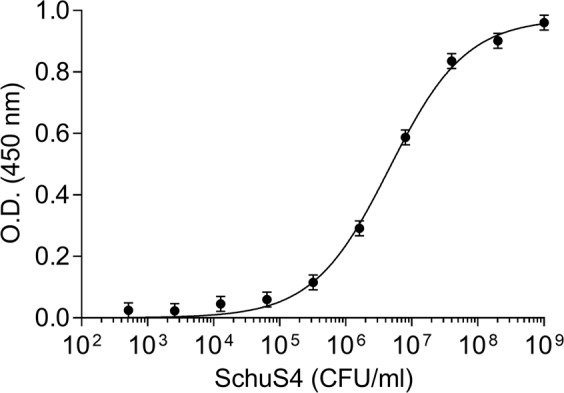
Detection of *Ft* by ELISA. SchuS4 at increasing concentrations was incubated in TL1-coated wells for one hour. The plate was then washed and HRP conjugated anti-*Ft* IgG antibodies were added. Points are average ± STD fitted by non-linear regression.

Early and sensitive detection of *Ft* is of high importance in order to initiate prompt life-saving antibiotic treatment^31^, several assays were introduced aiming for sensitive and specific detection of this agent^32–34^. The findings that TL1 exhibits high affinity and specificity toward *Ft* prompted us to examine its activity in a detection assay of the virulent SchuS4 (work was carried out under BSL-3 Class microbiological safety conditions). Thus, TL1 was immobilized on an ELISA plate to serve as the capture moiety, incubated with increasing concentrations (10^2^–10^9^ CFU/ml) of live SchuS4 and the IgG fraction of anti-*Ft* hyper-immune rabbit sera (termed T5) served as the detection component. Indeed, a sigmoidal dose response curve was generated with an estimated limit of detection (LOD) of 1 × 10^4^ CFU/ml (Fig. 4). We have previously demonstrated that using a novel assay that was based on biolayer interferometry (BLI) biosensor and the T5 antibody preparation, an LOD of 1 × 10^4^ CFU/ml was reached^35^. In comparison, the LOD values of commercially available detection kits for *Ft* are in the range of 1 × 10^5^ to 1 × 10^7^ CFU/ml^36^. It should be noted that by combining ELISA and gold-nanoparticles-linked oligonucleotide, an even lower sensitivity (1 × 10^3^ CFU/ml) could be reached^34^. Thus, it can be suggested that by incorporating TL1 in other assay formats that are more sensitive than the classical ELISA, further improvements in the detection of *Ft* may be achieved.

### Binding of TL1-scFv inhibits *F. tularensis* uptake by macrophages

**Figure 5:**
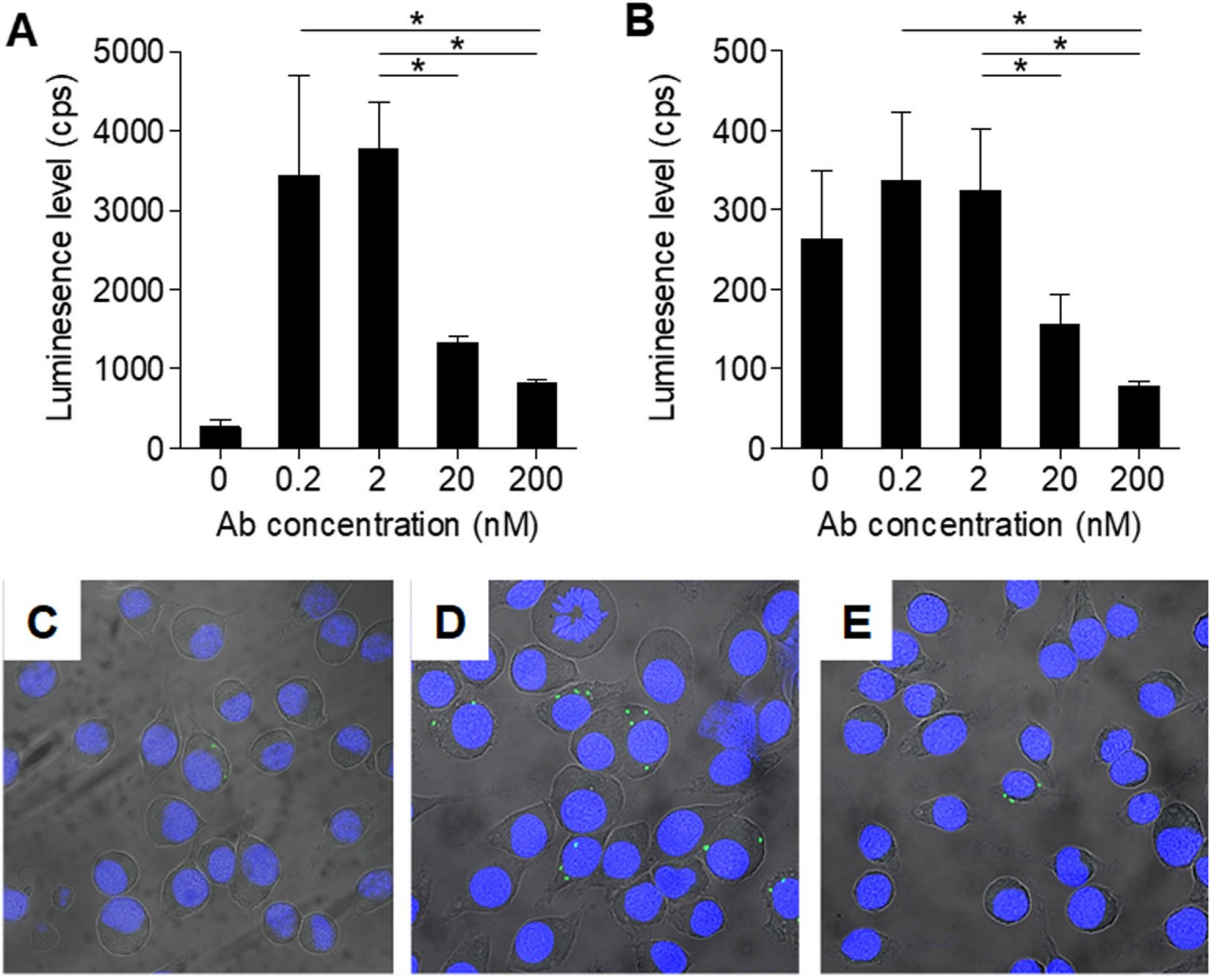
The effect of TL1 on *Ft* uptake by macrophages. Cultured J774A.1 murine macrophages were incubated with LVS-pXB173-lux in the absence (control) or in the presence of the indicated concentrations of either (**A**) TL1 or (**B**) TL1-scFv. The cells were lysed 24 hours later and the intracellular luminescence levels were determined. Bars are mean ± SEM of three independent experiments; *p < 0.05. (**C**–**E**) Macrophages were incubated with LVS (MOI = 1) in the absence (**C**) or in the presence of TL1 (200 nM) (**D**) or TL1-scFv (200 nM) (**E**) for two hours. The cells were then washed, and LVS bacterial cells were stained using an Alexa 488-conjugated rabbit anti-*Ft* antibodies. Cell nuclei were stained with DAPI.

Having such a potent anti *Ft*-LPS monoclonal antibody prompted us to ask whether the binding of this antibody will affect the uptake of *Ft* by macrophages. The experimental setup included cultured J774A.1 murine macrophages that were incubated with LVS-pXB173-lux that constitutively express luciferase. The cells were lysed 24 hours later and the intracellular luminescence levels were determined. In preliminary experiments it was found that there is a direct correlation between the bacterial LVS-pXB173-lux CFU (ranging from 1.4 × 10^4^ to 1 × 10^7^ CFU) and the luminescence levels (see Supplementary Fig. S1). Here, the cultured J774A.1 were incubated with LVS-pXB173-lux at MOI = 1 (1.23 × 10^5^ CFU), in the absence (control) or in the presence of TL1 and the intracellular luminescence levels were determined 24 hours later. As expected, incubation of LVS in the presence of TL1 (0.2 and 2 nM) significantly enhanced their uptake by 13–16 fold (Fig. 5A). Interestingly, at higher concentrations of TL1, the bacterial uptake level dramatically declined in an antibody-dose dependent manner (to 6 and 4-fold over control at 20 and 200 nM of TL1, respectively). These results are in line with previous observations that binding of antibody-coated bacteria to FcγR enhance bacterial-uptake by macrophages and neutrophils^9,18^. However, the fact that at higher concentrations the binding of the antibodies to *Ft*-LPS interferes with the phagocytosis process may suggest that two mechanisms co-exist (uptake-enhancement versus uptake-inhibition). It was therefore of interest to test the direct effect of antibody binding to *Ft*-LPS on bacterial uptake, while eliminating the FcγR mediated uptake.

We therefore created a soluble single-chain fragment (scFv) of TL1 (TL1-scFv) that comprises of the VH-VL regions of the antibody and lack the Fc region. Binding studies using octet revealed that the TL1-scFv retained its affinity toward *Ft* (exhibiting the same binding pattern as the IgG format; data not shown). Next, cultured macrophages were incubated with LVS-pXB173-lux in the presence of increasing concentrations of TL1-scFv and bacterial uptake was measured 24 hours later. Here, it was found that in the presence of 0.2 and 2 nM of TL1-scFv, there is no significant change in the amount of bacterial uptake when compared to control (Fig. 5B). However, increasing the TL1-scFv concentrations to 20 and 200 nM dramatically affected the bacterial uptake, where at 200 nM this process was inhibited by 70%.

Using qualitative confocal microscopy we also verified that the observed inhibitory effect of TL1-scFv is due to its effect on the direct interactions of LVS with the macrophages and not due to the bacteria’s inability to multiply intracellularly, To this end, murine macrophages were incubated with LVS and either TL1 or TL1-scFv for a short incubation period (2 hours) to limit the bacteria’s ability to multiply intracellularly, followed by fixation and imaging using polyclonal anti-LVS antibodies. Indeed, only in the presence of TL1 an increase of macrophages-containing bacteria was observed (Fig. 5C–E).

The role of antibodies in protection against *Ft* is not well understood. Several studies have demonstrated that passive administration of anti-*Ft* antibodies, and especially these that target the LPS can provide protection against LVS but not against the virulent SchuS4 type^9,11,37,38^. It was hypothesized that the main function of these antibodies is to enhance the bacterial clearance upon binding of the antibody-coated bacteria to FcγR on macrophages and neutrophils^9^. This route of bacterial clearance was also found to be complement-independent, and it was suggested that *Ft* is protected against complement-mediated killing through the expression of the O-antigen^9,39^. Moreover, the Fc-receptor targeting was also used to enhance the immune response toward *Ft* by administrating monoclonal antibody-inactivated *Ft* immune complexes^40^. On the other hand, both macrophages and neutrophils are the cell types that support *Ft* growth *in vivo*, therefore posing a possible paradox for the role of antibody-mediated opsonization of *Ft*.

In light of our recent findings, it is possible that the antibodies used as passive immunization to *Ft* infections in the literature vary in their dose, affinity and ability to bind the LPS moiety thereby providing conflicting results. For example, at lower doses the antibodies may enhance the bacterial uptake and thus hinder effective therapy, while in the cases where higher doses are implemented, the antibodies interfere with the ability of the bacteria to interact with macrophages and neutrophils thereby providing advantageous treatment. The results of this study may suggest that anti *Ft*-LPS that lack the ability to bind FcγR might provide a more uniformed outcome and inhibit bacterial uptake.

The applicability of this approach is not straight forward as it requires the production of engineered antibodies that contain a mutated Fc-region that lack the ability to bind FcγR while retaining their pharmacokinetics parameters^41^. Alternatively, one can use the scFv molecule as a passive therapy for *Ft* infection. However, since scFv’s have very short half-life in the circulation^42^, it should be taken into account when conceiving a study to test its effectiveness *in vivo*.

To conclude, we have isolated a specific anti *Ft*-LPS antibody that exhibits high affinity, thus making it an attractive candidate for incorporation in various detection systems for sensitive detection of the bacteria. Moreover, we have demonstrated that by eliminating its ability to bind the FcγR, binding of the antibody-derivative to the bacterial surface inhibits it uptake by macrophages *in vitro*. These results may promote further experiments to test whether this effect will provide better therapeutic efficacy *in vivo*, alone or in combination with antibiotic treatment.

## Materials and Methods

### Bacterial and cell cultures

*Francisella tularensis subsp*. *tularensis* (SchuS4) strain and *Francisella tularensis* subsp. *holarctica* strain LVS were grown as described before^43^. Work with SchuS4 was carried out under BSL-3 Class microbiological safety conditions. *Francisella fillomeragia* strains (ATCC25015, ATCC25017) were grown as described earlier for the LVS stain^43^. The bioluminescence reporter plasmid pXB173-lux was generously obtained from James E. Bina^44^ and introduced into wild-type *F*. *tularensis* LVS, resulting in constitutive bioluminescence production. In order to increase the bioluminescent signal, the original gro promoter was replaced with the bfr promoter, which has been found to be more potent^45^. The resulting LVS-pXB173-lux was grown in TSBC broth (0.1% L-cysteine, 3% tryptic soy broth) or CHA agar (1% hemoglobin, 5.1% Cysteine heart agar) supplemented with 2 µg/ml chloramphenicol (Cm).

J774A.1 murine macrophage like cells were obtained from the American Type Culture Collection (ATCC, BALB/C macrophage). The cells were grown in flasks in Dulbecco’s Modified Eagle Medium (DMEM, Biological Industries, Beit Haemek, Israel) supplemented with 10% fetal bovine serum (FBS), 2 mM L-glutamine and 1 mM sodium pyruvate and maintained at 37 °C in a humidified 5% CO_2_ incubator.

LPS was purified from LVS bacteria as described before^46^. LVS was inactivated by exposure of 5 × 10^9^ CFU/ml to 3 doses of 75,000 µj/cm3 UV radiation. SchuS4 was inactivated by boiling approximately 8.5 × 10^10^ CFU/ml in 2 × Laemmli sample buffer (Bio-Rad, USA) for 30 min.

### Rabbit immunization

Animals were treated according to the regulations outlined in the U.S. Department of Agriculture (USDA) Animal Welfare Act and the conditions specified in the Guide for Care and Use of Laboratory Animals (National Institute of Health, 2011). The immunization study (RB-15-2013) was approved by the “Israel Institute for Biological Research Institutional Ethical Committee on Animal Experiments”. Female New Zealand White (NZW) *Oryctolagus cuniculus* (rabbit) was immunized by live LVS strain injected at 6 consecutive sub-cutanic injections over a period of 24 weeks. The first four injections consisted of 1 ml of 1 × 10^8^ CFU that were given monthly, with the exception of the 4^th^ injection that was given two months after the 3^rd^ injection. The next two monthly booster injections consisted of 1 × 10^9^ CFU. Seven days after the last boost, the rabbit was sacrificed and samples were taken from its blood and lymphatic nodes for library construction, as described before^19^.

### Antibodies

Anti *F*. *tularensis* polyclonal IgG fraction (designated T5) was obtained by HiTrap Protein A chromatography (GE Healthcare, Uppsala, Sweden) of the hyper-immune rabbit serum immunized as described in the previous section.

### scFv Library construction and screening

RNA extracted from spleen, bone marrow and blood samples was used as a template for first-strand cDNA synthesis and a set of degenerate primers was used to amplify all known sequences of *Oryctolagus cuniculus* VH and Vk immunoglobulin families^19^. All other methods for the construction and screening of the library were essentially as described before^19^ with the following changes: Inactivated LVS were used (1 × 10^8^ cfu/mL in carbonate-bicarbonate buffer) to coat a polystyrene immuno-tube (Nunc, Denmark). The bacterial solution was then removed, followed by the tube baing blocked (2% SM + 0.05% Tween 20 in PBS) and used for the next panning steps. Single colonies were randomly picked from the third panning output, and phages were rescued and tested for their binding to LVS bacteria.

### ELISA

Direct ELISA: All steps were performed essentially as described before^19^ with the following changes: Plates were coated with 2 × 10^8^ CFU/mL of inactivated LVS in Carbonate bicarbonate buffer (Sigma-Aldrich, St. Louis, MO, USA). Individual phage clones, antibodies or rabbit sera were added to the plates for a one-hour incubation; the plates were then washed with PBST and incubated with the detecting antibody: horseradish peroxidase (HRP)-conjugated anti-M13 antibody (GE healthcare, Little Chalfont, UK) for phage clones, anti-human IgG conjugated to alkaline phosphatase (Jackson immunoresearch, West Grove, PA, USA) for full antibodies or anti-rabbit conjugated to alkaline phosphatase (Sigma-Aldrich, St. Louis, MO, USA) for serum ELISA. Detection of HRP conjugates was achieved with 3,3′,5,5′-tetramethybenzidine (TMB/E, Millipore, Billerica, MA, USA) while detection of alkaline phosphatase conjugates was achieved with SIGMAFAST p-nitrophenyl phosphate tablets (Sigma-Aldrich, St. Louis, MO, USA).

Capture ELISA: Plates were coated overnight with 2 µg/ml TL1 antibody in Carbonate bicarbonate buffer. Live SchuS4 bacteria (work was carried out under BSL-3 Class microbiological safety conditions) were diluted in PBS and added at different concentrations to the plates for a one-hour incubation; the plates were then washed with PBST and incubated with HRP conjugated T5^35^. The rest of the steps were as described for direct ELISA.

### Production of Full-Length antibodies

VH and VL sequences of the TL1 antibody clone were cloned into a mammalian full-length immunoglobulin expression vector^29^ resulting in IgG1/*κ* chimeric rabbit-human antibody expression. The vector was expressed in FreeStyle Max 293 cells (Thermo Scientific, Waltham, MA, USA) by transient transfection and the antibody was purified on a HiTrap Protein-A column. Purified TL1 antibody is now available from Sigma-Aldrich, USA (#SAB4200833).

### Antibody labeling

Biotinylation of the purified IgG antibody was carried out using sulfo-NHS-SS-biotin (sulfosuccinimidyl-2-(biotinamido) ethyl-1,3-dithiopropionate; Pierce; USA) according to the manufacturer’s instructions. Alkaline-phosphatase labeling of antibodies was carried out using the Lightning-link alkaline phosphatase conjugation kit (Innova Biosciences, UK). Conjugation of TL1 to Alexa488 was carried out using a commercial kit (Thermo fisher Scientific, USA) according to the manufacturer’s instruction.

### Construction and purification of soluble scFv TL1

The pET SUMO plasmid, part of the ChampionTM pET SUMO protein expression system (Invitrogen, USA), was used for cloning of TL1-scFv antibody for soluble expression. The scFv was amplified from phagemid DNA and cloned into linearized pET SUMO using A/T ligation. The plasmid was freshly transformed to *E*. *coli* BL21 (DE3) (Novagen, USA) and expression was carried out in Terrific Broth medium supplemented with 1% glucose and 50 µg/mL Kanamycin at 37 °C, 250 rpm. When the suspension reached an OD_600_ of 0.7–0.9, IPTG was added to a final concentration of 0.5 mM and the temperature was lowered to 25 °C. After an O.N growth the cells were harvested, re-suspended in 20 mM phosphate buffer pH 7.4 (supplemented with 0.5 M NaCl, 20 mM Imidazole and Protease inhibitors; Sigma-Aldrich, USA) and sonicated under ice. After sonication, the suspension was precipitated (9500 g, 20 minutes, 4 °C) and Benzonase nuclease (Sigma-Aldrich, USA) was added to a final concentration of 50 units/mL to the supernatant. The supernatant was then filtered (45 µm) and the SUMO-scFv was purified on a HisTrap column (GE, USA) according to the manufacturer’s instructions. The buffer of the purified antibody was exchanged to PBS using a 10 Kd Amicon ultra (Millipore, USA).

### Binding studies

Binding studies were carried out using the Octet Red system (ForteBio, USA) essentially as described before^19^ using LVS bacteria or LPS extract^46^. The Octet data analysis software 8.1 (Fortebio, USA) was applied for sensorgrams fitting with a 1:1 binding model. The presented values are an average of several repeated measurements.

### Western blot

To obtain bacteria lysate, inactivated bacteria was boiled for 10 min with 4xLaemmli sample buffer (Bio-Rad, USA). Bacterial lysates, LPS and protein markers (Precision Plus protein standards dual color; Bio-Rad, USA) were resolved on NuPAGE 4–12% Bis-Tris gel 1.5 mm × 10well (Invitrogen, USA). Gels were blotted on a nitrocellulose filter (iBlot NC gel transfer stacks, Mini; Invitrogen, USA) and blocked for 1 hour in Odyssey blocking buffer (Li-Cor, USA). The nitrocellulose filters were then washed 3 times in wash solution (1% 10 mM Tris 1 M pH 8, 3% NaCl 5 M, 0.05% Tween20 in 1 liter dH_2_O) and then probed (4 °C, O.N) with T5 or TL1 that were diluted in incubation buffer (5% nonfat dry milk; Bio-Rad, USA). The nitrocellulose filters were then washed 3 times in wash solution. T5 was detected with Goat anti rabbit IRDye 800 CW (Li-Cor, USA) diluted 1:20,000 in incubation buffer. TL1 was detected with Goat anti human IRDye 800 CW (Li-Cor, USA) diluted 1:20,000 in incubation buffer. After another extensive wash step the filters were developed in ODYSSEY CLx (Li-Cor, USA).

### IFA

IFA was carried out with LVS bacteria (1 × 10^8^ cfu/ml) air dried on a multispot slide. The slide was incubated for 30 min (37 °C, humid incubator) with Alexa 488 conjugated TL1, diluted to a final concentration of 1 µg/ml in assay buffer (PBS supplemented with 2% BSA and 0.05% tween20). Following incubation the slide was rinsed with water and dried. The slide was than examined under fluorescent illumination with a Nikon phase microscope (Nikon eclipse E400).

### Macrophage infection assay

J774A.1 macrophages were seeded at 2 × 10^4^ cells/well in white 96 well plates (Corning, Corning, NY), and allowed to adhere overnight. On the next day, logarithmic phase LVS-pXB173-lux bacteria were washed twice with PBS and incubated with 0.2, 2, 20 or 200 nM of TL1 or scFv-TL1 for 1 hour at room temperature. Bacteria were added to the macrophages at an MOI (multiplicity of infection) of 1, and the plate was then centrifuged at 1000 rpm for 5 minutes. After 1 hour incubation at 37 °C in a humidified 5% CO2 incubator, cells were washed twice with PBS and gentamicin (2 µg/ml) was added to the growth medium for 24 hours, after which the luminescence level was evaluated using the Victor3 (Perkin Elmer) luminometer. Statistical analyses (p value) were performed using Prism software (Version 5.01, GraphPad Software Inc., La Jolla, CA, USA, 2007) in an unpaired two-tailed t-test.

For the confocal microscopy, J774A.1 cells were seeded on 8-well chamber slides (ibidi, Martinsried, Germany) at 1 × 10^5^ cells/well, and allowed to adhere overnight. Cells were then infected as described above for 2 hours, washed three times with PBS and fixed in ice-cold 100% methanol for 2 minutes. Cells were blocked in PBS + 2% BSA + 2% naïve rabbit serum for 20 minutes in 37 °C. Bacteria were stained using an Alexa 488-conjugated rabbit anti-*F*. *tularensis* serum (1:200). Cell nuclei were stained with DAPI (1μg/ml, Sigma-Aldrich, St. Louis, MO, USA). Samples were viewed using a Zeiss LSM710 confocal microscope (Zeiss, Oberkochen, Germany).

-No datasets were generated or analyzed during the current study.

## Supplementary information

Supplemental 1
