# Supplementary material for: Inhibition of Francisella tularensis phagocytosis using a novel anti-LPS scFv antibody fragment
Source: Sci Rep. 2019 Aug 6;9:11418. doi: 10.1038/s41598-019-47931-w (PMC6684794; doi:10.1038/s41598-019-47931-w)
Supplement: Supplementary file 1 — Supplemental 1 [file 41598_2019_47931_MOESM1_ESM.pdf]

Inhibition of *Francisella tularensis* phagocytosis using a novel anti-LPS scFv antibody fragment

Adva Mechaly[a], Uri Elia[b], Ron Alcalay[b], Hila Cohen[b], Eyal Epstein[c], Ofer Cohen[b*] and Ohad Mazor[a*]

The Departments of Infectious Diseases[a], Biochemistry and Molecular Genetics[b] and Biotechnology[c] ; Israel Institute for Biological Research, Ness-Ziona, Israel

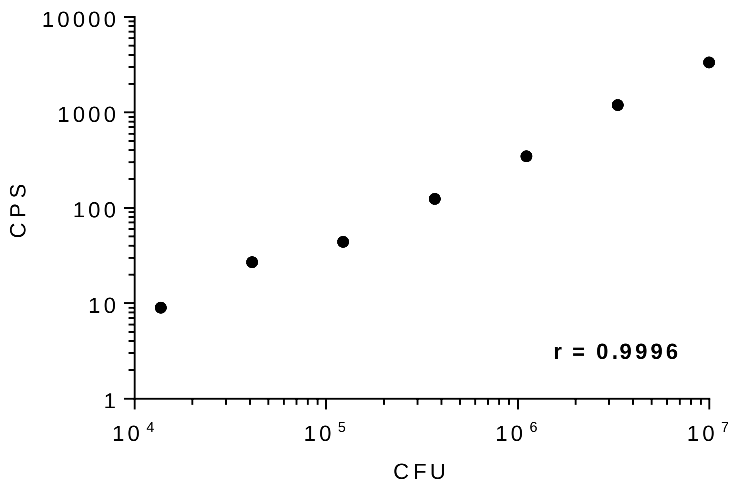

Supplementary Figure S1. Correlation between the bacterial LVS-pXB173-lux CFU (ranging from $1.4x10^4$ to $1x10^7$ CFU) and the luminescence levels.
